# A novel hypoxia-driven gene signature that can predict the prognosis and drug resistance of gliomas

**DOI:** 10.3389/fgene.2022.976356

**Published:** 2022-09-02

**Authors:** Peng Ren, Jing-Ya Wang, Zhi-Rui Zeng, Nan-Xi Li, Hong-Lei Chen, Xin-Ge Peng, Ujjal K. Bhawal, Wen-Zhi Guo

**Affiliations:** ^1^ Department of Anesthesiology, The Seventh Medical Center of Chinese PLA General Hospital, Beijing, China; ^2^ Guizhou Provincial Key Laboratory of Pathogenesis and Drug Research on Common Chronic Diseases, Department of Physiology, School of Basic Medical Sciences, Guizhou Medical University, Guizhou, China; ^3^ Department of Pharmaceutical Sciences, Beijing Institute of Radiation Medicine, Beijing, China; ^4^ Hengyang Medical College, University of South China, Hengyang, China; ^5^ Department of Biochemistry and Molecular Biology, Nihon University School of Dentistry at Matsudo, Chiba, Japan; ^6^ Department of Pharmacology, Saveetha Dental College, Saveetha Institute of Medical and Technical Sciences, Chennai, India

**Keywords:** glioma, hypoxia-driven gene, prognostic prediction, drug resistance gene, SHOX2, MGMT

## Abstract

Hypoxia spontaneously forms in the interior of glioma tissues and regulates the expression of various genes. However, the status of hypoxia-driven genes in glioma tissues is not completely known. In the current study, RNA-seq data of 695 glioma tissues in The Cancer Genome Atlas (TCGA) were set as a discovery cohort and were used to identify hypoxia-driven genes and construct a novel gene signature. The prognostic values of that signature were verified in data from the TCGA and the Chinese Glioma Genome Atlas (CGGA). The expression and diagnostic values of hypoxia-driven genes were analyzed using immunohistochemistry and receiver operator characteristic curves. Finally, the effects of hypoxia-driven genes on temozolomide (TMZ) resistance were analyzed by western blot, CCK-8 and colony formation assay. A total of 169 hypoxia-driven genes were identified, which were associated with a poor outcome in glioma patients. Among them, 22 genes had a degree score ≥10 and 6 genes (WT1, HOXA2, HOXC6, MMP9, SHOX2 and MYOD1) were selected to construct a signature to classify glioma patients into low- or high-risk groups. That signature had a remarkable prognostic value for glioma patients in TCGA and CGGA. The expression of HOXC6, MMP9, SHOX2 and MYOD1 was associated with hypoxia degree in glioma tissues and in recurrent cases, had a remarkable diagnostic value and a significant relationship with disease free survival in glioma patients. Moreover, SHOX2 was highly expressed in glioma tissues with O-6-methylguanine-DNA methyltransferase (MGMT)-unmethylation and temozolomide (TMZ) resistant glioma cell lines, and associated with MGMT expression. Knockdown the expression of SHOX2 significantly reduced the TMZ-resistance induced by hypoxia in glioma cells. Ultimately, we identified six novel hypoxia-driven genes for reliable prognostic prediction in gliomas and found that SHOX2 might be a potential target to overcome the TMZ resistance induced by hypoxia.

## Introduction

Gliomas are the most common and aggressive primary central nervous system tumors in adults (Mitchell et al., 2022; Wang et al., 2022). Despite significant improvements in surgical treatment and the arrival of the chemo-therapeutic drug temozolomide (TMZ), the prognosis of patients with malignant gliomas is still dismal (Ren et al., 2021; Wang et al., 2022). Hypoxia is one of the important characteristics of the tumor microenvironment (Li et al., 2021). The progression in malignancy of gliomas is associated with an increase in the number of cells and a poorly organized tumor vascular system, leading to an inadequate blood supply, hypoxia areas, and eventually to necrosis, which is characteristic of gliomas (Amberger-Murphy,2009; Liu et al., 2020). Hypoxic tumors are more resistant to treatment with chemotherapy and/or radiation resulting in lower overall survival of various tumor types including gliomas (Li et al., 2021). Therefore, the identification of hypoxia-driven genes may contribute to the therapy of gliomas.

Previous studies indicated that a hypoxia microenvironment is an important driving force promoting the expression of proto-oncogenes in tumor cell lines, as well as cell proliferation, angiogenesis and metastasis (Bhandari et al., 2019; Sharma et al., 2022; Tang et al., 2022). In addition, cancer cells have diverse mechanisms of therapy resistance, which are either intrinsic to tumor development or acquired because of extrinsic factors (Gao et al., 2021; Goenka et al., 2021). Among them, a hypoxic condition is one of the most important contributors to chemotherapy resistance due to the lack of vasculature, drug perfusion is difficult, which leads to an unfavorable prognosis (Goenka et al., 2021). Meanwhile, hypoxia as an microenvironmental condition can activate the transcriptional activity of a protein called hypoxia-inducible factor (HIF) (Li et al., 2022). HIF binds to an enhancer element known as the hypoxia response element (HRE), which activates the transcription of over 40 hypoxia-responsive genes to promote progenesis, invasion, resistance and recurrence (Ao et al., 2021). Until now, a series of target genes of HIF has been identified, including BCL2/adenovirus E1B protein interacting protein 3, vascular endothelial growth factor and programmed death ligand-1 (Ding et al., 2021; Wu et al., 2019). As hypoxia-driven genes, their expression is elevated in glioma tissues which is positively correlated with a poor prognosis (Ding et al., 2021; Du et al., 2017; Seyedmirzaei et al., 2021). However, the role and effects of hypoxia-driven genes in gliomas are still largely un-known, especially the hypoxia-driven genes associated with drug resistance.

This study aimed to identify hypoxia-driven genes in gliomas and establish a predictive risk model that could provide valuable insights for the prognostic prediction of glioma patients, as well as explore potentially effective targets for overcoming hypoxia and TMZ resistance.

## Materials and methods

### Data acquisition and preprocessing

The RNA-seq profiles and corresponding clinical characteristics of gliomas were downloaded from The Cancer Genome Atlas (TCGA; https://portal.gdc.cancer.gov) and the Chinese Glioma Genome Atlas (CGGA; http://www.cgga.org.cn/), respectively. A total of 695 glioma patients in the TCGA and 325 glioma patients in the CCGA were enrolled in this study, and were set as training and test cohorts, respectively. Before performing the analysis, the data matrix in the TCGA and CGGA was normalized, logarithmically processed and the IDs were converted into gene symbols using R software (Version: 4.1.2).

### Identification of hypoxia characteristics in glioma tissues

We first downloaded the gene set of 50 genes involved in the hypoxia form Gene Set Enrichment Analysis (GSEA) database (http://www.gsea-msigdb.org/gsea/index.jsp) using the index phrase “BUF-FA_HYPOXIA_METAGENE”. We then obtained the gene expression patterns of those 50 genes in each glioma tissue from the RNA-seq profiles of TCGA, and calculated the hypoxia signature score for each sample using the t-distribution random neighbor embedding method. Finally, the degree of hypoxia in tumor tissues was determined by the hypoxic signature score, and the 695 glioma tissues from TCGA database were grouped (high or low hypoxia groups) according to their hypoxic signature scores. The detailed scores of enrolled glioma tissues in TCGA are listed in Supplementary Table S1.

### Survival analysis

The relevance of overall survival (OS) and disease-free survival (DFS) rates in the two groups was determined by Kaplan–Meier curve analysis using R package com-ponents. A *p* < 0.05 was set as the cut-off to consider statistically significant differences.

### Analysis of DEGs

EdgeR package in R software was used to analyze DEGs comparing two groups of individuals. DEGs (|LogFC| >1 and adjusted *p* values < 0.05) were identified and plotted in volcano plots and in heatmaps.

### GSEA analysis

Patients were categorized into two groups of high or low expression, according to the median values of gene expression. GSEA software analysis was used to ascertain the hallmark pathways enriched. Normalized enrichment scores (NES) were calculated and adjusted for *p*-values, and terms with *p* < 0.05 were considered statistically significant, with the top 5 NES visualized.

### Protein-protein interaction network

The original PPI network was constructed in the STRING database (https://cn.string-db.org/) *via* importing the information of genes. The information of the original PPI network was exported into a text file and visualized using Cytoscape software. The connectivities between genes were calculated and are represented as degree scores. Genes with degree scores ≥10 were set as hub genes in the PPI network.

### Prognostic gene screening and prognostic signature

The Survival package was used to perform the univariate Cox regression analysis as well as the Forestplot package to obtain *p*-values, HRs and 95% CIs. A threshold of *p* < 0.05 was used to screen candidate prognostic genes. Further screening of the candidate prognostic genes was performed by LASSO Cox regression. Briefly, features were selected using the LASSO regression algorithm, and parameters were determined by 10-fold cross-validation. The genes obtained from LASSO regression were calculated to construct the risk score equation and the prognostic risk score of each patient was calculated based on the parameters of the risk model, after which the patients were subcategorized into high-risk or low-risk groups on the basis of their median risk scores. To evaluate the clinical value of the risk model, Kaplan-Meier survival analysis was performed for the two groups, and receiver operating characteristic (ROC) curves were used to evaluate its sensitivity and specificity.

### Clinical sample collection

Glioma tissues from 60 patients were obtained from the Department of Neuro-surgery, Seventh Medical Center of Chinese PLA General Hospital, Beijing China. Among them, 42 were primary gliomas and 18 were recurrent gliomas. Two pathologists independently diagnosed enrolled patients with gliomas based on the WHO Classification of Tumors of the Central Nervous System. Prior to sample collection, none of the patients had undergone radiotherapy or chemotherapy. The glioma tissues were collected and stored at −80°C before performing further experiments. In-formed consent was obtained from all patients, and the Human Ethics Committee of Seventh Medical Center of Chinese PLA General Hospital (*Approval number: 2022-43*) approved the collection and use of tissues.

### Immunohistochemistry staining

The 60 glioma tissues embedded in paraffin were dewaxed and dehydrated with xylene and an ethanol gradient, respectively. Antigen retrieval (10× Citrate Buffer, pH 6.0, Sigma-Aldrich, United States) was performed for specimens and peroxidase blocking (Servicebio, Wuhan, China)) was carried out, followed by incubation overnight at 4°C with primary antibodies including hypoxyprobe pimonidazole (combined with Mab; 1:200; Cat no. HP1-100 kit; Hypoxyprobe, Inc., United States), HOXC6 (1:8000; Cat no. PA5-41479, Thermo Fisher Scientific, United States), MMP9 (1:500; Cat No. 10375-2-AP, Proteintech, Wuhan, China), WT1 (1:200; Cat No. A16319, Abconal, Wuhan, China), SHOX2 (1:200; Cat No. ab229851, Abcam, United States), MYOD1 (1:500; Cat No. 18943-1-AP, Proteintech, Wuhan, China) and HOXA2 (1:200; Cat No. ab229960, Abcam, United States). After three rounds of washing with PBS, the same DAB staining conditions were performed on each specimen after incubation with the secondary antibody (Boster, Wuhan, China). Additionally, antigen-antibody complex signaling within glioma tissues was observed using an orthotopic light microscope.

### TMZ sensitivity analysis

OncoPredict is an R package contributed by Danielle Maeser et al. (Maeser et al., 2021) for predicting *in vivo* or cancer patient drug response. It fits the gene expression profile of the tissues to the half maximal inhibitory concentration (IC50) of the cancer cell lines to drug from Genomics of Drug Sensitivity in Cancer (GDSC; https://www.cancerrxgene.org/) and the gene expression profile of cancer lines from Broad Institute Cancer Cell Line Encyclopedia (CCLE; https://portals.broadinstitute.org/ccle_legacy/home). Through OncoPredict, drug score of each tissues can be accessed, while high drug score means increased IC50 in the tissues.

### Cell culture and transfection

The glioma cell lines U87, A172 and T98G were obtained from Procell (Wuhan, China), while SF268, LN-18 and U373 was purchased from Otwo Biotech (Shenzhen, China). All cell lines were cultured in the DMEM medium contained 10% FBS in the 37°C environment with 5% CO_2_. The hypoxia environment were simulated in a three-air incubator (Thermo Fisher Scientific, United States) with the parameter of 94% N_2_, 1% O_2_ and 5% CO_2_. The small interfering RNA (siRNA) targeting SHOX2 (si-SHOX2) and scramble siRNA were obtained from iGene Biotechnology Co., Ltd. (Beijing, China). The transfection of siRNAs were conducted via Lipo2000 reagent (Sangon, Shanghai, China). The sequences of si-SHOX2 and scramble were 5′-GGA​CCA​ATT​TCA​CCC​TGG​AAC-3′ and 5′-GTT​CTC​CGA​ACG​TGT​CAC​GT-3′.

### Cell count kit-8

Glioma cells were set into a 96 well plate with a density of 5000 cells/well. While cells were adhered, different concentration of TMZ were added for 48 h. Following by removing medium, 90 μl DMEM mixed with 10 μl CCK-8 reagent were added into per well for 2 h, and the absorbance of each wells were determined in multimode reader (Biorad, United States).

### Colony formation assay

Glioma cells were set into plates (radius = 3 cm) with a density of 2000 cells/plate. While cells were adhered, TMZ (20 μM) was added and cells were cultured under hypoxia and normal condition for 12 days. Then, the cell colonies were immobilized with 0.5% paraformaldehyde (Servicebio, Beijing, China) for 1 h, and stained with 1% crystal violet for 1 h. After washing, the cell colony were recorded and counted.

### Western blot

Total protein in glioma cells were obtained via RIPA reagent (Servicebio, Beijing, China), while its concentration was determined using BCA method. After conducting electrophoresis in SDS-PAGE (Meilune, Dalian, China), protein was transferred into PVDF membranes (Thermo Fisher Scientific, United States). After blocking in 5% skim milk, primary antibodies anti-SHOX2 (1:1000, Cat No. 16366-1-AP; Proteintech, Wuhan, China) and anti-ACTB (1:10,000, Cat No. AC026; Abconal, Wuhan, China) were added for 14 h. Then, the membranes were washed by TBST and incubated by second antibodies. The membranes were finally developed via ECL reagent (Proteintech, Wuhan, China).

### Statistical analysis

All statistical analyses were performed using R software (version 4.0.0). An unpaired *t*-test or one-way ANOVA with Tukey’s test was used to determine differences between groups. A *p* < 0.05 was considered statistically significant.

## Results

### Analysis of the landscape between high-hypoxia and low-hypoxia glioma tissues

**FIGURE 1:**
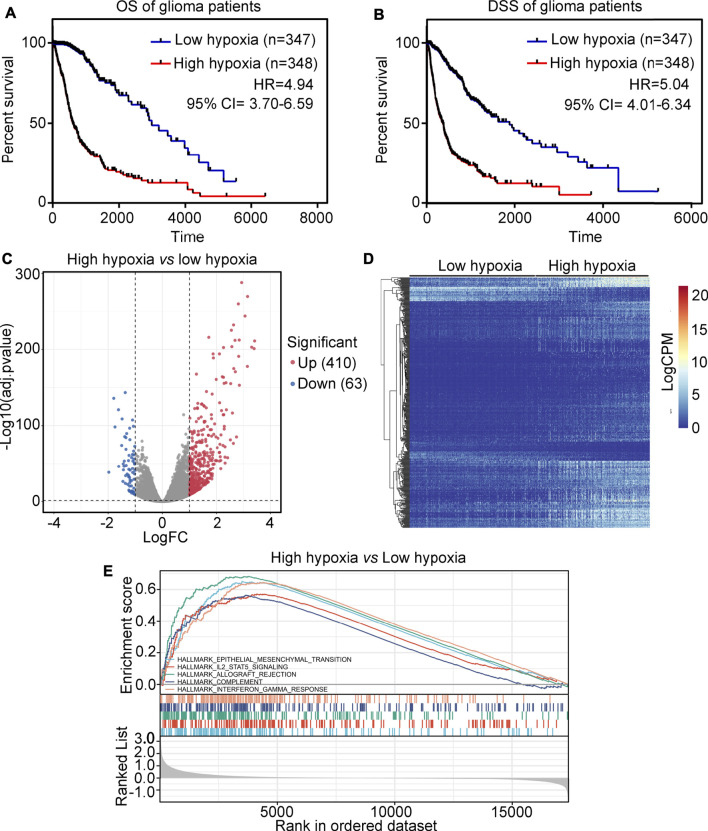
Analysis of the landscape between high-hypoxia and low-hypoxia glioma tissues. **(A)** The overall survival (OS) rate between high-hypoxia and low-hypoxia groups of glioma patients. **(B)** The disease-specific survival (DSS) rate between high-hypoxia and low-hypoxia groups of glioma patients. **(C)** Volcano plot indicating DEGs between high-hypoxia and low-hypoxia groups of glioma tissues. **(D)** Heatmap indicating DEGs between high-hypoxia and low-hypoxia group of glioma tissues. **(E)** GSEA analysis of terms enriched in DEGs between the high-hypoxia and low-hypoxia groups of glioma tissues.

The glioma tissues in the TCGA were divided into two groups, high-hypoxia or low-hypoxia, based on their hypoxic signatures. Kaplan survival analysis showed that patients in the high-hypoxia group had a lower overall survival (OS) rate (HR = 4.94, Figure 1A) and disease specific survival (DSS) rate (HR = 5.04, Figure 1B) compared to patients in the low-hypoxia group. To perform analysis of differentially expressed genes (DEGs), a total of 410 up-regulated genes and 63 down-regulated genes were identified between the high-hypoxia and low-hypoxia groups of glioma tissues (Figures 1C,D). DEGs for high- and for low-hypoxia glioma tissues were determined to be hypoxia-driven genes. A GSEA study revealed that hypoxia in glioma was positively associated with epithelial-mesenchymal transition, IL2-STAT5 signaling, allograft rejection, complement and interferon-*γ* response (Figure 1E).

### Screening of important hypoxia-driven genes associated with poor outcome in glioma tissues

**FIGURE 2:**
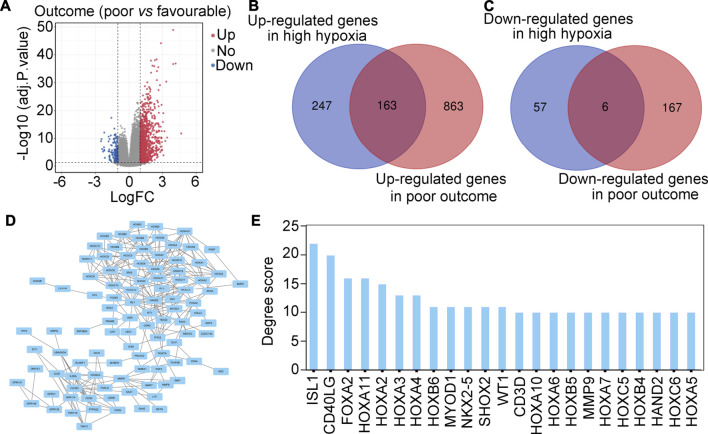
Screening important hypoxia-driven genes associated with poor outcome in glioma tis-sues. **(A)** Volcano plot indicating DEGs between glioma patients with poor (progressive disease, PD) or favorable (complete response, CR + partial response, PR + stable disease, SD) outcomes. **(B)** Intersection analysis demonstrated that 163 genes were up-regulated in gliomas with high-hypoxia and poor outcome. **(C)** Intersection analysis demonstrated that 6 genes were down-regulated in gliomas with high hypoxia and poor outcome. **(D)** PPI network of 169 important hypoxia driven genes associated with outcome in glioma. **(E)** Degree score of genes in the PPI network ≥10.

We then determined whether these hypoxia-driven genes were associated with poor outcome. Analysis of DEGs was performed, and a total of 1,026 up-regulated genes and 173 down-regulated genes between glioma patients with poor (progressive disease, PD) or favorable outcomes (complete response, CR + partial response, PR + stable disease, SD) were found (Figure 2A). Intersection analysis was then performed and identified 163 hypoxia-driven genes that were highly expressed in glioma tissues and were positively associated with poor outcome (Figure 2B). A total of 6 hypoxia-driven genes were expressed at low levels in glioma patients, and were negatively associated with poor outcome (Figure 2C). Moreover, these 169 hypoxia-driven genes were used to construct a PPI network (Figure 2D), and the degree score in this PPI network was analyzed. Finally, a total of 23 genes with degree scores ≥10 was set as the important hypoxia-driven genes (Figure 2E).

### Modeling of prognostic risk in the training cohort

**FIGURE 3:**
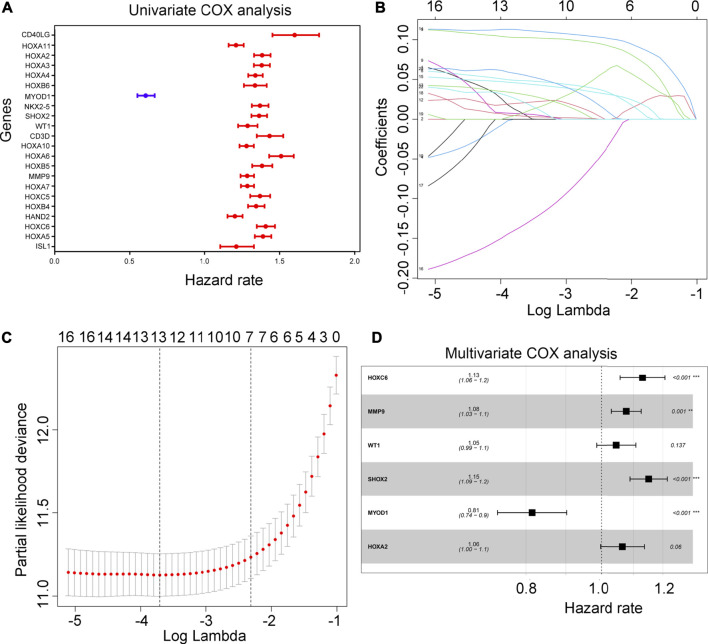
Construction of risk model. **(A)** Univariate COX analysis for the 22 important hypoxia-driven genes. Blue indicates genes that act as predictors of a favorable prognosis, while red indicates genes that act as predictors of an unfavorable prognosis. **(B,C)** LASSO penalized Cox regression analysis exhibiting the 13 important hypoxia-driven genes. **(D)** Multivariate COX regression analysis selected 6 important hypoxia-driven genes to construct the risk model. HOXC6, MMP9. SHOX2 and MYOD1 can act as an independent prognostic factor.

Using univariate Cox regression analysis, 21 hypoxia-driven genes were associated with an unfavorable prognosis in the TCGA, yet 1 hypoxia-driven gene was as-sociated with a favorable prognosis (Figure 3A). Following LASSO penalized cox regression analysis, we identified 13 important hypoxia-driven genes, including ISL1, HOXC6, MMP9, HOXB5, CD3D, WT1, SHOX2, NKX2-5, MYOD1, HOXA4, HOXA3, HOXA2 and CD40LG (Figures 3B,C). Moreover, after performing multivariate COX regression analysis, WT1, HOXA2, HOXC6, MMP9, SHOX2 and MYOD1 were selected to establish a predictive signature for glioma patients in the training cohort TCGA with a risk score = (0.044 × WT1 expression) + (0.062 × HOXA2 expression) + (0.122 × HOXC6 expression) + (0.074 × MMP9 expression) + (0.140 × SHOX2 expression) + (-0.205× MYOD1 expression). Interesting, through multivariate COX regression analysis, we found that HOXC6, MMP9, SHOX2 and MYOD1 can act as independent prognostic factors for glioma (Figure 3D).

### Prognostic risk model validation in the TCGA training cohort

**FIGURE 4:**
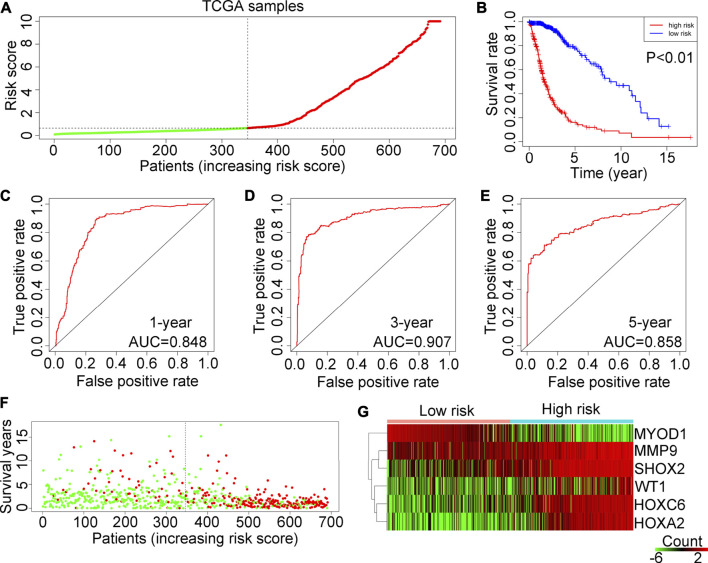
Examination of the risk model in the TCGA training cohort. **(A)** Glioma samples in the TCGA were divided into low-risk and high-risk groups. **(B)** Kaplan–Meier plot analysis of the overall survival rates of low-risk or high-risk patients with glioma in the TCGA. **(C–E)** Receiver operating characteristic (ROC) curves showing the diagnostic value of the risk model for the 1-year, 3-year and 5-year survival rates of patients with gliomas in the TCGA. **(F)** Survival time and status of each glioma patient in the TCGA cohort. **(G)** Expression levels of MYOD1, MMP9, SHOX2, WT1, HOXC6 and HOXA2 in glioma tissues from patients with high-risk or low-risk scores in the TCGA.

The patients in the TCGA training cohort were categorized into two groups (high-risk or low-risk) based on the median risk score (Figure 4A). The results demonstrated that glioma patients in the training cohort TCGA with a high-risk score had shorter OS rates (Figure 4B). According to ROC analysis, the AUCs for predicting 1-year, 3-year and 5-year survival rates in TCGA glioma patients were 0.848, 0.907 and 0.858, respectively (Figures 4C–E). In addition, more deaths were observed in glioma patients with high-risk scores in the TCGA (Figure 4F). Heatmap analysis showed that WT1, HOXA2, HOXC6, MMP9 and SHOX2 are highly expressed in high-hypoxia glioma tissues in the TCGA, whereas MYOD1 expression is reduced (Figure 4G).

### Prognostic risk model validation in the test cohort CGGA

**FIGURE 5:**
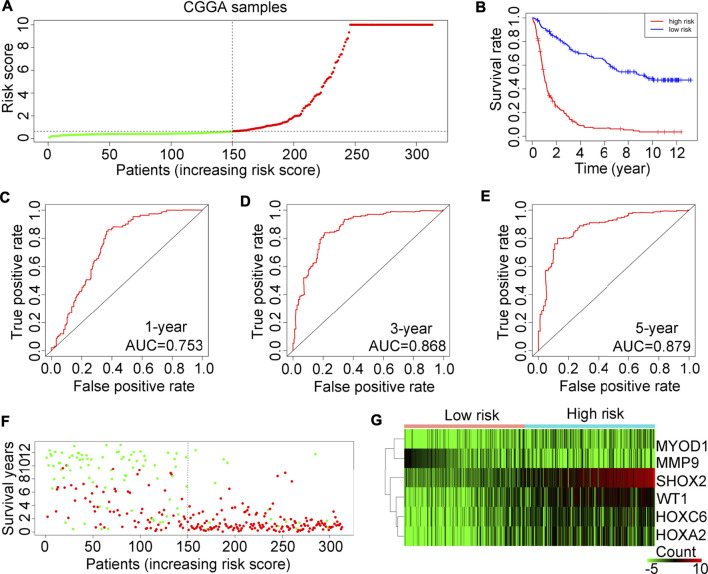
Examination of the risk model in the CGGA training cohort. **(A)** Glioma samples in the CGGA were divided into low-risk or high-risk groups. **(B)** Kaplan-Meier plot analysis of the overall survival rate in low-risk and in high-risk patients with glioma in the CGGA. **(C–E)** Receiver operating characteristic (ROC) curves showing the diagnostic value of the risk model for the 1-year, 3-year and 5-year survival rates of patients with gliomas in the CGGA. **(F)** Survival time and status of each glioma patient in the CGGA cohort. **(G)** Expression levels of MYOD1, MMP9, SHOX2, WT1, HOXC6 and HOXA2 in glioma tissues from patients with high-risk or low-risk scores in the CGGA.

The prognostic risk model applicability was tested on 325 glioma tissues in the CGGA database. In accordance with the prognostic risk model preliminarily estimated in the TCGA, glioma tissues in the test cohort CGGA were also divided into high or low-risk score groups (Figure 5A). Consistent with findings in the TCGA, glioma patients with high-risk scores also had a lower OS rate (Figure 5B). In the test cohort CGGA, AUCs for 1-year, 3-year and 5-year survival rates were 0.753, 0.868 and 0.879, respectively (Figures 5C–E). In line with the results in the TCGA training cohort, we found that the high-risk score group of glioma patients in the test cohort CGGA had a higher death rate (Figure 5F). Also, in the test cohort of CGGA, the expression of SHOX2, WT1, HOXA2 and HOXC6 was elevated (Figure 5G).

### HOXC6, MMP9, SHOX2 and MYOD1 are differentially expressed in primary and in Re-current glioma tissues, and are associated with hypoxia

**FIGURE 6:**
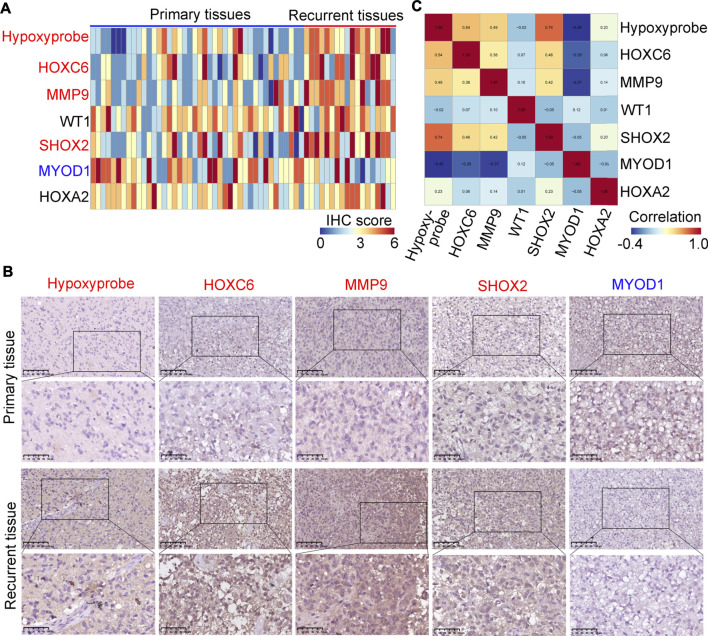
Verification of the expression levels of MYOD1, MMP9, SHOX2, WT1, HOXC6 and HOXA2 in glioma tissues. **(A)** Expression of hypoxyprobe, HOXC6, MMP9, WT1, SHOX2, MYOD1 and HOXA2 in each glioma tissue (*n* = 60). **(B)** Representative IHC stained images of Hypoxyprobe, HOXC6, MMP9, SHOX2 and MYOD1 in primary and/or recurrent glioma tissues (compared to primary glioma tissues, red gene names in recurrent tissues indicate high expression and blue gene names indicate reduced expression). **(C)** Co-expressed score of Hypoxyprobe, HOXC6, MMP9, WT1, SHOX2, MYOD1 and HOXA2 in gliomas.

We used a hypoxyprobe to detect the hypoxia level in 42 primary and in 18 re-current glioma tissues. It was demonstrated that recurrent tissues had higher hypoxic levels (Figures 6A,B). Similarly, we found that the expression of HOXC6, MMP9 and SHOX2 was higher in recurrent glioma tissues compared with primary glioma tissues and the expression of MYOD1 was decreased (Figures 6A,B), while there were no significant changes in WT1 and HOXA2 (Supplementary Figure S1). Then, we performed co-expression analysis for HOXC6, MMP9, SHOX2 and hypoxia level according to the IHC score in each sample, we found that the expression of HOXC6, MMP9 and SHOX2 was positively associated with the hypoxic level, while the expression of MYOD1 was negatively associated with the hypoxic level (Figure 6C). Moreover, through performing ROC analysis, we found that HOXC6, MMP9, SHOX2 and MYOD1 had a high diagnostic value (AUC>0.7) to distinguish primary and recurrent glioma tissues (Supplementary Figure S2). Furthermore, results analyzed in glioma tissues in the TCGA demonstrated that glioma patients with high expression levels of HOXC6, MMP9 and SHOX2 had lower dis-ease-free survival rates. Similarly, patients with low expression levels of MYOD1 also had lower disease-free survival rates (Supplementary Figure S3). These results indicate that HOXC6, MMP9, SHOX2 and MYOD1 may be involved in the recurrence of gliomas.

### SHOX2 is associated with TMZ-resistance

**FIGURE 7:**
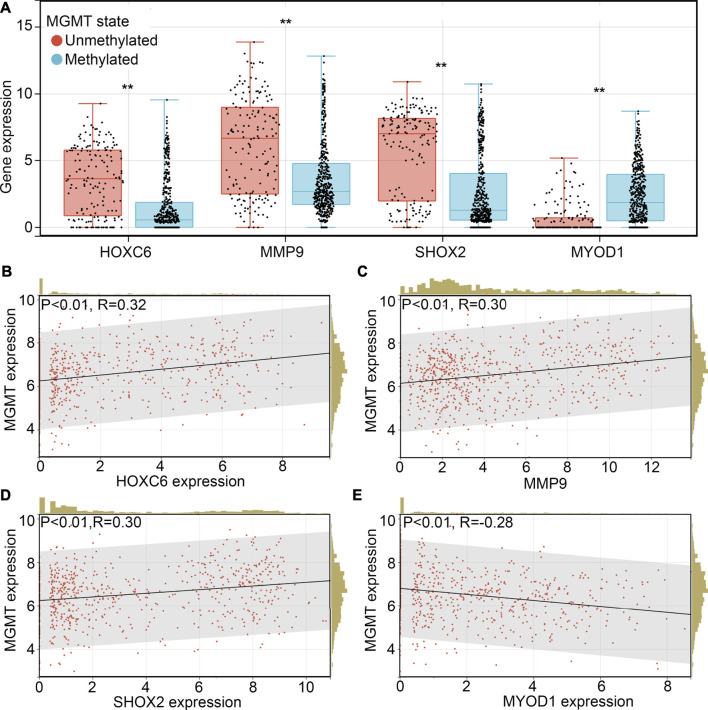
SHOX2 was associated with MGMT expression. **(A)** Expression of HOXC6, MMP9, SHOX2 and MYOD1 in glioma tissues with promoter methylation and promoter unmethylation. Co-expression relationship between HOXC6 **(B)**, MMP9 **(C)**, SHOX2 **(D)** and MYOD1 **(E)** between MGMT in glioma tissues. **, *p* < 0.01.

**FIGURE 8:**
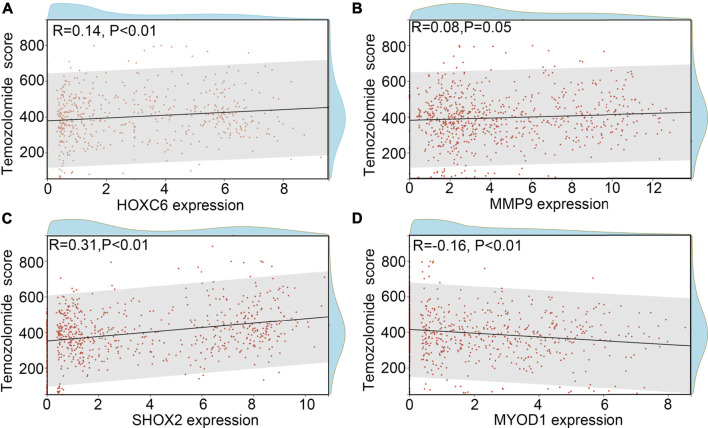
SHOX2 was associated with TMZ-score. **(A)** Relationship between HOXC6 and TMZ-score. **(B)** Relationship between MMP9 and TMZ-score. **(C)** Relationship between SHOX2 and TMZ-score. **(D)** Relationship between MYOD1 and TMZ-score.

TMZ-resistance is a key factor which allows the recurrence of glioma. Therefore, we wanted to determine whether HOXC6, MMP9, SHOX2 and/or MYOD1 is involved in the TMZ-resistance. Previous studies indicated that promoter methylation of O-6-methylguanine-DNA methyltransferase (MGMT), a key member involved in the DNA repair, would increase the sensitivity of glioma cells or tissues to TMZ (Oldrini et al., 2020). We first determined the expression HOXC6, MMP9, SHOX2 and MYOD1 in glioma tissues with promoter-methylation and promoter-unmethylation of MGMT. Higher expression levels of HOXC6, MMP9 and SHOX2, and lower expression levels of MYOD1 were observed in the glioma tissues with promoter-unmethylation of MGMT in TCGA (Figure 7A). Similarly, through performing co-expression analysis, we found that expression of HOXC6 (Figure 7B), MMP9 (Figure 7C) and SHOX2 (Figure 7D) were also positively associated with MGMT expression, while MYOD1 expression (Figure 7E) was negatively associated with MGMT expression. Moreover, after calculating TMZ score via OncoPredict, we found that expression of HOXC6 (Figure 8A), MMP9 (Figure 8B) and MYOD1 (Figure 8D) had no significant relationship with TMZ score, while SHOX2 expression (Figure 8C) had positively relationship with TMZ score. These evidences indicated that hypoxia-related gene SHOX2 may involve in the resistance of TMZ.

### SHOX2 was key mediator linking to hypoxia and TMZ-resistance

**FIGURE 9:**
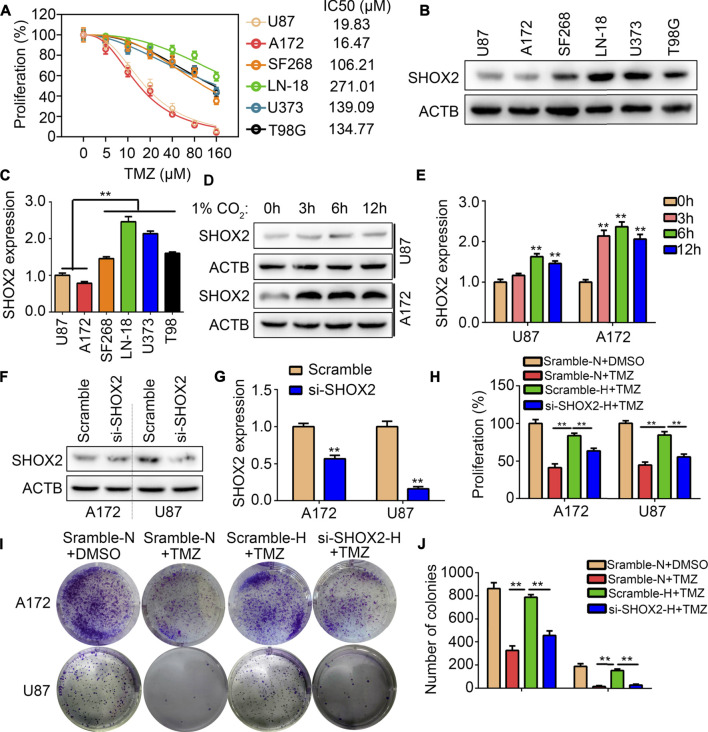
SHOX2 linked to hypoxia and TMZ-resistance. **(A)** CCK-8 was used to determine the IC50 of TMZ in 48 h for U87, A172, SF268, LN-18, U373 and T98 cells. **(B,C)** Western blot was used to determine the expression of SHOX2 in U87, A172, SF268, LN-18, U373 and T98 cells. **(D,E)** Western blot was used to determine the expression of SHOX2 in U87 and A172 cells under hypoxia for 0, 3, 6 and 12 h **(F,G)** Western blotting was to determine the expression of SHOX2 in U87 and A172 after treatment with siRNAs. **(H)** CCK-8 assays were used to determine the effects of SHOX2 on TMZ-resistance under hypoxia. **(I,J)** Colony formation assays were used to determine the effects of SHOX2 on TMZ-resistance under hypoxia. **, *p* < 0.01.

We analyzed the IC50 of 48 h in the glioma cells U87, U172, SF268, LN-18, U373 and T98G through CCK-8 method. It was demonstrated that the IC50 of 48 h in U87, U172, SF268, LN-18, U373 and T98G were 19.83, 16.47, 106.21, 271.01, 139.09 and 134.77 μM (Figure 9A). We then analyzed the expression of SHOX2 in TMZ-sensitivity cells (U87 and A172) and TMZ-resistance cells (SF268, LN-18, U373 and T98G). It was demonstrated that expression of SHOX2 was significantly elevated in TMZ-resistance cells (Figures 9B,C). We further cultured TMZ-sensitivity cells U87 and A172 under hypoxia, and found that SHOX2 was significantly increased (Figures 9D,E). To verify whether SHOX2 linked hypoxia and TMZ-resistance, we used targeting siRNA to inhibit the expression of SHOX2 in U87 and A172 cells (Figures 9F,G). CCK-8 results indicated that hypoxia would blocked the inhibitory effects of TMZ (20 μM) in U87 and A172 cells, while knockdown of SHOX2 could abolish the effects of hypoxia (Figure 9H). Similarly, colony formation assays indicated that hypoxia relieved the inhibitory effects of TMZ (20 μM) on U87 and A172 cells, while suppression of SHOX2 blocked the effects of hypoxia (Figures 9I,J). These evidences verified that SHOX2 linked hypoxia and TMZ-resistance.

### GSEA for exploring the regulated mechanism of SHOX2 in glioma

**FIGURE 10:**
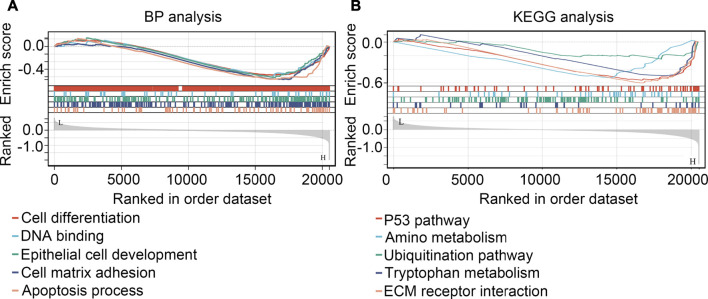
GSEA analysis for the biological process terms (BP, **(A)** and KEGG terms **(B)** affected by SHOX2.

GSEA were performed for preliminarily exploring the regulated mechanism of SHOX2 in glioma. It was demonstrated that SHOX2 had potential to inhibit the cell differentiation, DNA binding, epithelial cell development, cell matrix adhesion and apoptosis (BP terms; Figure 10A). For signaling pathway, SHOX2 had potential to inhibit the P53 pathway, amino metabolism, ubiquitination pathway, tryptophan metabolism and ECM receptor interaction (KEGG terms, Figure 10B).

## Discussion

Hypoxia is a very important characteristic associated with solid tumor microenvironments *in vivo*. Hypoxia-driven glioma stem cells are an important basis for glioma therapeutic resistance due to their intrinsic properties and adaptive resistance pathway (Osuka and Van Meir, 2017). A series of experimental studies has shown that hypoxia can shape and maintain the phenotype of glioma stem cells (Yang et al., 2012), driving therapeutic resistance and recurrence of gliomas and eventually leading to a poor prognosis (Boyd et al., 2021; Yang et al., 2012). In addition, hypoxia elicits metabolic alterations *via* the up-regulation of key metabolic enzymes that help them adapt to changes in nutrient requirements and redox status (Maremonti et al., 2020), and contribute to resistance of existing glioma therapies (Bao and Wong, 2021). Overcoming drug resistance in hypoxic gliomas may be an available avenue to improve patients’ quality of life. Thus, identifying hypoxia-driven genes may uncover the molecular mechanism(s) that underlies hypoxic responses in gliomas, as well as provide potential biomarkers for the diagnosis and therapeutic targets of the disease.

In the present study, consistent with previous studies, we found that glioma tissues with a high hypoxic signature had lower OS and DSS. A total of 473 DEGs between low-hypoxia and high-hypoxia groups was identified. Subsequent univariate or multivariate COX regression analyses eventually constructed a six hypoxia-driven gene signature related to a prognostic risk model, which was found to have a remarkable prognostic value for glioma patients in the TCGA, and was also suitable for patients in the CGGA. IHC staining of primary and recurrent glioma tissues suggested that the expression of HOXC6, MMP9, MYOD1 and SHOX2 are associated with the degree of hypoxia in gliomas as well in recurrent cases. Interestingly, results from glioma tissues in the TCGA also indicated that patients with high expression levels of HOXC6, MY-OD1 and SHOX2, and low expression levels of MYOD1, had a lower disease-free survival rate.

HOXC6 is a member of the homeobox C cluster, which is comprised of 9 vital family members located on chromosome 12 in tandem (Alvarado et al., 2016). HOXC6 is reportedly over-expressed and acts as an oncogenic gene in many types of malignancies (Alvarado et al., 2016). Upregulated HOXC6 is an independent risk factor and predicts poor prognosis in glioma patients (Yu et al., 2021). MMP9 is a matricellular protein associated with extracellular matrix remodeling that regulates the activity of cell adhesion molecules and cytokines and promotes tumor progression (Mehner et al., 2014). In hypoxic conditions, hypoxia promotes the invasion of retinoblastoma HXO-RB44 cells by activating the HIF-1α/MMP9 signaling pathway (Li and Zheng, 2017). In breast cancer, elevated expression of MMP9 is a predictor of shorter patient survival (Joseph et al., 2020). Consistent with our study of gliomas, MMP9 was identified as a hypoxia-driven gene associated with a poor outcome. MYOD1 is a transcription factor that promotes the expression of muscle-specific genes and a high expression level of MYOD1 inhibits cell renewal, promotes terminal differentiation and induces apoptosis (Wu et al., 2020). MYOD1 has been reported to be expressed and to be functionally abnormal in a variety of tumor types (Peng et al., 2016; Wu et al., 2020; Zhang et al., 2017). In gastric cancer, MYOD1 expression was significantly decreased, and suppresses the migration and invasion of gastric cancer cells by inhibiting FUT4 transcription (Wu et al., 2020). SHOX2 is a key transcriptional regulator in several genetic diseases, and SHOX2 has been shown to be a valuable biomarker in the diagnosis and evaluation of many types of cancers (Kneip et al., 2011; Teng et al., 2021), including gliomas (Zhang et al., 2019,^2016^). Zhang et al. reported that in lower grade glioma patients, SHOX2 expression has been found to be a predictive indicator of survival and has been used as an independent indicator (Zhang et al., 2016). Likewise, another study revealed that SHOX2 could also serve as a potential indicator for tumor treatment with a prognostic value for gliomas (Zhang et al., 2019). Constant with previous studies, the risk constructed by these four genes had high prognosis value. Furthermore, our present study provided the first evidences that dysregulation of these four genes might driving by hypoxia.

A combination of TMZ and radiation therapy is currently the standard treatment for patients with glioma. TMZ, a prodrug alkylating agent, adds a methyl group to purine and pyrimidine in DNA, thus leading to the death of glioma cells (Karachi et al., 2018). However, due to MGMT had potential to repair the damage DNA, 55% glioma had resistance to TMZ (Wick et al., 2014). In the present study, through perform IHC, we found that high expression of HOXC6, MMP9 and SHOX2 and low expression of MYOD1 were observed in recurrent glioma tissues. Therefore, we considered that whether these four genes involved in the TMZ-resistance. Previous studies indicated that the promoter methylation of MGMT would reduced the transcription of MGMT in glioma cells, thus increasing the sensitivity to TMZ (Oldrini et al., 2020). Interesting, in the current study, we found that HOXC6, MMP9 and SHOX2 expression were increased in the glioma tissues with promoter unmethylation of MGMT, while MYOD1 was reduced. Previous study indicated that MGMT can be regulated by HIF1α, a direct and key hypoxia response element, under hypoxic or normoxic environment (Tang et al., 2016; Vlachostergios et al., 2013). As HOCX6, MMP9, SHOX2 and MYOD1 were hypoxia-associated genes, we then determined their co-expression relationship. We found that all of these four genes were associated with MGMT expression in glioma tissues. Especially, we found SHOX2 expression was associated with TMZ-score. These evidences indicated that hypoxia associated gene SHOX2 may involve in the TMZ-resistance.

Previous studies indicated that hypoxia can regulated a series oncogenes (Liu et al., 2020) or non-coding RNAs (Ho et al., 2022) to induce TMZ-resistance of glioma. In the current study, we demonstrated that SHOX2 was increased TMZ-resistance glioma cells, while hypoxia can induced the elevation of SHOX2 in glioma cells. Hypoxia increased the resistance of glioma cells, and suppression of SHOX2 could abolish the effects of hypoxia. These may be the first evidences to demonstrated that SHOX2 linked to hypoxia and TMZ-resistance. Finally, to study the mechanism of SHOX2 in glioma cells, GSEA was performed, and a series of pathways were found inhibiting by SHOX2, especially for P53 pathway. Previous studies indicated that P53 can bind to the promoter of MGMT genes, and reduced its expression and increased the sensitivity of glioma cells to TMZ (Tsai et al., 2021). Furthermore, a research in lung cancer showed that SHOX2 promotes tumorigenesis in lung cancer cells through the down-regulation of p53 and the activation of NF-κB, and leads to drug resistance in lung cancer cells through the aberrant regulation of cell cycle progression and the inhibition of apoptosis (Yang et al., 2015). Therefore, we speculated that SHOX2 may suppress the P53 and increased the MGMT expression, thus inducing TMZ-resistance.

There are some limitations in our present study. First, the speculation that SOX2 affected TMZ-resistance via P53/MGMT axis should be verified by more experiments. Then, the effects of SOX2 *in vivo* should be determined in animal experiments. Furthermore, more experiments need to perform to determine whether other mechanisms of SHOX2 involved in TMZ-resistance independed of transcription factor, as GSEA predicating that it can regulate ubiquitination.

In conclusion, a risk model constructed by 6 hypoxia-driven genes (WT1, HOXA2, HOXC6, MMP9, SHOX2 and MYOD1) provide valuable clinical utility for the prognostic prediction of glioma patients. Importantly, SHOX2 might be a potential target for blocking the stimulative effects of a hypoxic environment and TMZ resistance in glioma progression.

## Data Availability

The original contributions presented in the study are included in the article/Supplementary Material, further inquiries can be directed to the corresponding authors.
