# Supplementary figures and images for: A novel hypoxia-driven gene signature that can predict the prognosis and drug resistance of gliomas
Source: Front Genet. 2022 Sep 2;13:976356. doi: 10.3389/fgene.2022.976356 (PMC9478203; doi:10.3389/fgene.2022.976356)

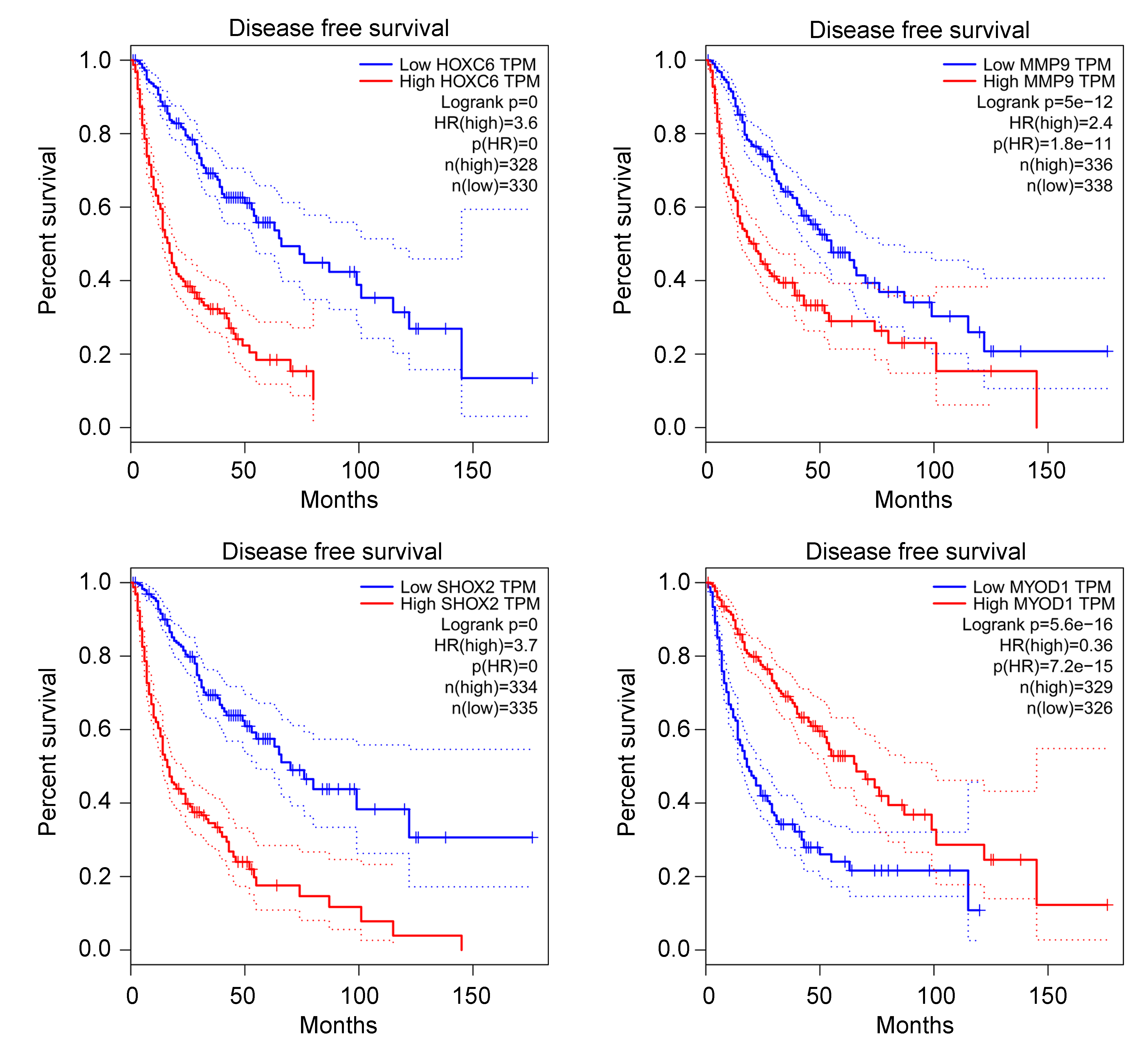

Supplement: Supplementary file 1 [file Image3.TIF]

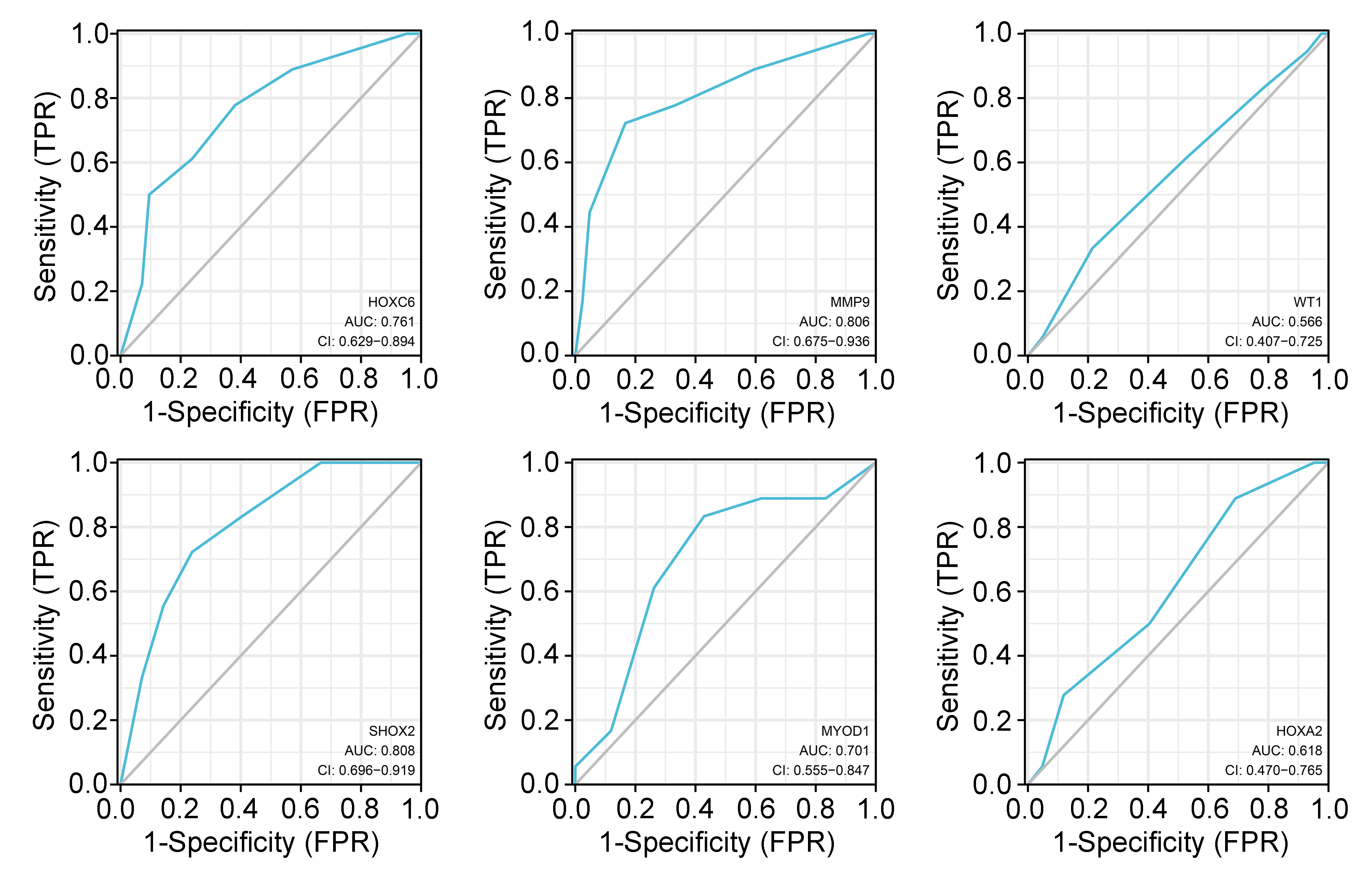

Supplement: Supplementary file 2 [file Image2.TIF]

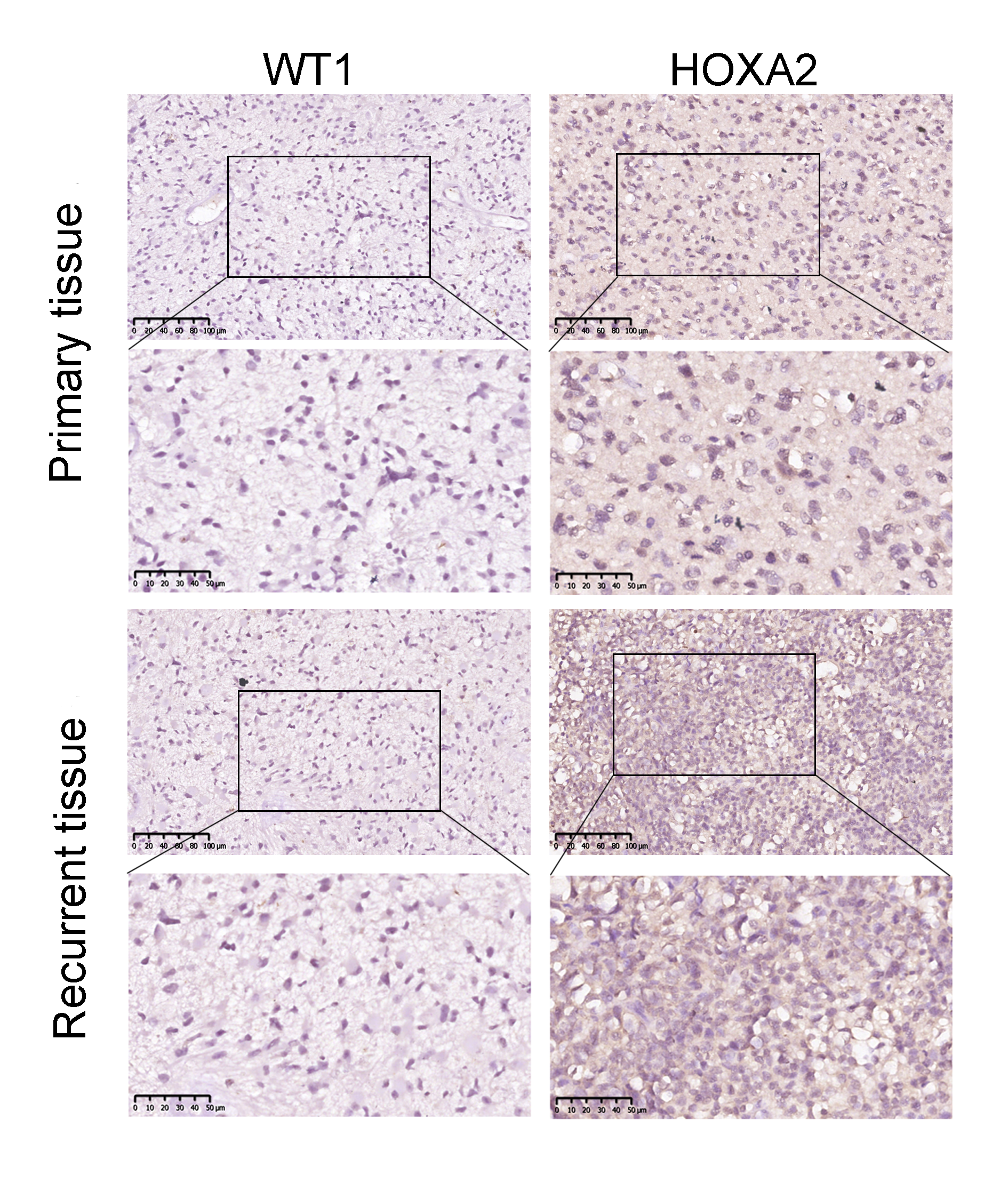

Supplement: Supplementary file 3 [file Image1.TIF]
